# Reduction of apoptosis and preservation of mitochondrial integrity under ischemia/reperfusion injury is mediated by estrogen receptor β

**DOI:** 10.1186/s13293-016-0104-8

**Published:** 2016-09-23

**Authors:** Carola Schubert, Valeria Raparelli, Christina Westphal, Elke Dworatzek, George Petrov, Georgios Kararigas, Vera Regitz-Zagrosek

**Affiliations:** 1 Institute of Gender in Medicine & Center for Cardiovascular Research, Charité-Universitaetsmedizin, Hessische Str. 3-4, 10115 Berlin, Germany; 2 DZHK (German Center for Cardiovascular Research), partner site Berlin, Berlin, Germany; 3 Department of Experimental Medicine, Sapienza University of Rome, Rome, Italy; 4 Max-Delbrück-Center for Molecular Medicine Berlin-Buch, Berlin, Germany; 5 Klinik für Kardiovaskuläre Chirurgie, Universitätsklinikum Düsseldorf, Düsseldorf, Germany

**Keywords:** Apoptosis, Estrogen receptor β, Ischemia/reperfusion, Mitochondria, Myosin light chain

## Abstract

**Background:**

Estrogen improves cardiac recovery after ischemia/reperfusion (I/R) by yet incompletely understood mechanisms. Mitochondria play a crucial role in I/R injury through cytochrome c-dependent apoptosis activation. We tested the hypothesis that 17β-estradiol (E2) as well as a specific ERβ agonist improve cardiac recovery through estrogen receptor (ER)β-mediated mechanisms by reducing mitochondria-induced apoptosis and preserving mitochondrial integrity.

**Methods:**

We randomized ovariectomized C57BL/6N mice 24h before I/R to pre-treatment with E2 or a specific ERβ agonist (ERβA). Isolated hearts were perfused for 20min prior to 30min global ischemia followed by 40min reperfusion.

**Results:**

Compared with controls, ERβA and E2 treated groups showed a significant improvement in cardiac recovery, i.e. an increase in left ventricular developed pressure, dP/dtmax and dP/dtmin. ERβA and E2 pre-treatment led to a significant reduction in apoptosis with decreased cytochrome c release from the mitochondria and increased mitochondrial levels of anti-apoptotic Bcl2 and ACAA2. Protein levels of mitochondrial translocase inner membrane (TIM23) and mitochondrial complex I of respiratory chain were increased by ERβA and E2 pre-treatment. Furthermore, we found a significant increase of myosin light chain 2 (MLC2) phosphorylation together with ERK1/2 activation in E2, but not in ERβA treated groups.

**Conclusions:**

Activation of ERβ is essential for the improvement of cardiac recovery after I/R through the inhibition of apoptosis and preservation of mitochondrial integrity and can be a achieved by a specific ERβ agonist. Furthermore, E2 modulates MLC2 activation after I/R independent of ERβ.

**Electronic supplementary material:**

The online version of this article (doi:10.1186/s13293-016-0104-8) contains supplementary material, which is available to authorized users.

## Background

Premenopausal women have a decreased risk of ischemic heart disease compared with age-matched men, suggesting a putative cardioprotective effect related to estrogens during reproductive age. However, in randomized control trials, chronic estrogen therapy in postmenopausal women has not demonstrated readily any cardiovascular benefit [1, 2]. To clarify these conflicting clinical data, more mechanistic studies in animal models are needed. There is ample evidence that 17β-estradiol (E2) protects from ischemia/reperfusion (I/R) injury in mice [3–7]. Effects of E2 are mainly mediated by estrogen receptors (ER) α or β in the heart. Which ER protects the female myocardium under I/R conditions is not fully understood. The role of ERβ is of particular interest, since it has no significant effects in the reproductive system and ERβ agonists are being developed for clinical use in different indications [8].

To study the effects of I/R in rodents, generally either myocardial infarction (MI) with subsequent reperfusion or isolated perfused heart systems with global or regional ischemia, i.e., the Langendorff model, have been used [9]. In the Langendorff model, there is convincing evidence that hearts of ovariectomized (ovx) mice exhibit a significantly greater degree of I/R injury than hearts from intact female mice, suggesting that E2 protects from I/R injury [5, 7, 10]. The administration of E2 reduced infarct size [5, 11, 12] or enhanced functional recovery in the Langendorff model under I/R conditions [5, 13, 14]. Some studies suggested a predominant role of ERβ mediating cardioprotection in females [4, 5, 7, 15], yet the mechanism is not fully understood. Compared with WT or ERαKO, ERβKO female hearts exhibited less functional recovery after I/R [4]. In accordance with these findings, ovx mice treated for 2 weeks with E2 or the ERβ-specific agonist DPN prior to I/R exhibited significant better functional recovery compared to vehicle-treated ovx females [7]. Gene expression profiling demonstrated that E2 effects were mediated by ERβ activation resulting in upregulation of a number of protective genes involved in apoptosis/cell death regulation or in stress-activated kinases [7]. Considering the crucial role of mitochondria in cell survival [16], we hypothesized that the E2-induced improvement of cardiac recovery might be attributed to reduction of apoptosis-mediated myocardial damage through preservation of mitochondrial integrity by ERβ activation [17] and tested this hypothesis using a specific ERβ agonist in parallel with E2 in a Langendorff model in female ovx mice.

I/R injury has also been shown to lead to myofibrillar remodeling and to depress Ca^2+^ sensitivity of myofilaments. Myosin light chain 2 (MLC2) plays an important role in the regulation of muscle contractile activity via its phosphorylation [18, 19]. Monasky et al. reported that phosphorylation of MLC2 was significantly reduced in female WT hearts after I/R compared to baseline [20]. We found recently that E2 controls MLC2 function and thereby contractility in mice [21]. Therefore, the modulation of MLC2 activation in cardiac tissue by E2 after I/R and the role of ERβ deserve attention for the identification of protective mechanisms and were included in the present study.

## Methods

### Animals

Experiments were performed in accordance with the guidelines of Charité-Universitaetsmedizin, were approved by the Landesamt für Gesundheit und Soziales (LaGeSo, Berlin, Germany; G0216/12), and conform to the Guide for the Care and Use of Laboratory Animals published by the US National Institutes of health (NIH Publication No. 85-23, revised 1996). Female C57BL/6N mice (Janvier) were bilaterally ovariectomized (ovx) at the age of 10 weeks and kept on soy-free diet (Ssniff, Soest) for 4 weeks. Before I/R experiments, animals were randomized into three groups [vehicle treated (untreated) or treated either with E2 (0.2 mg/kg body weight (BW)) [21] or the specific ERβ agonist compound A (ERβA) (1.6 mg/kg BW; Karobio, Sweden) [22] in vivo by single i.p. injection 24 h before I/R].

### Langendorff heart preparation

Animals were anesthetized with ketamine hydrochloride (80 mg/ml)/xylazine hydrochloride (12 mg/ml) solution administered by intraperitoneal injection at a dose of 1 mg/kg BW and anticoagulated with heparin. The hearts were quickly excised and placed in ice-cold Krebs–Henseleit buffer containing in millimoles per liter: 2.1 MgSO_4_, 118.0 NaCl, 4.7 KCl, 0.06 EDTA, 24.7 NaHCO_3_, 0.23 KH_2_PO_4_, 3.215 CaCl_2_, and 10.0 glucose. The aorta was quickly cannulated for retrograde perfusion at a constant pressure of 80 mmHg using a Langendorff perfusion apparatus. The apparatus was water-jacketed to maintain a temperature of the heart at 37 °C. The buffer was oxygenated with 95 % oxygen/5 % CO_2_ to maintain a pH of 7.4. A water-filled latex balloon that was connected to a pressure transducer was inserted into the left ventricle (LV). LV diastolic pressure (LVPdia) was set to 12 mmHg. Heart rate (HR), LV systolic (LVPsys) and LV developed pressure (LVPdp), as well as dP/dtmin and dP/dtmax, markers for relaxation and contractility, were measured and recorded continuously using ISOHEART® Isolated Heart Data Acquisition Software (Harvard Apparatus, Massachusetts, USA). The hearts were perfused for a 20-min stabilization period. Following 30 min of global ischemia, the hearts were reperfused for a total of 40 min. Recovery of cardiac function was measured at the end of 40 min of reperfusion and expressed as a percentage of the rate prior to ischemia. During the Langendorff experiment, the physiological data were monitored continuously and calculated at the end of I/R in percent of the value at the start of ischemia.

### Determination of necrosis

For determination of necrosis, lactate dehydrogenase (LDH) was used as biomarker. Effluents of each group were collected for LDH assay over 3 min directly before global ischemia and four times over 3 min immediately after the start of reperfusion. LDH was assayed with the use of a commercially available assay (l-lactate dehydrogenase, Roche Applied Science, Mannheim, Germany) according to the manufacturer’s instructions. Optical density was measured in duplicate for each sample and multiplied by the respective coronary flow.

### Apoptosis detection

Frozen tissue from the left ventricles were cut into slides of 5 μm. Terminal deoxynucleotidyl transferase dUTP nick end labeling (TUNEL Boehringer, Mannheim, Germany) method was used to evaluate apoptosis in ischemic reperfused heart tissue. Staining was analyzed by Fluorescent Microscopy (Leica, 40-fold resolution). Results are expressed according to manufacturer indication in apoptotic nuclei percentage. Representative pictures are shown in Fig. 3.

### Western blot and fractioning of the heart

Whole cell extracts were isolated using modified RIPA buffer and separated by SDS-polyacrylamide gel electrophoresis as previously described [22]. The left ventricles were homogenized in a modified RIPA buffer (50 mM Tris, pH 7.4, 150 mM NaCl, 1 mM EDTA, 1 % NP-40, 0.25 % Na-deoxycholate) supplemented with protease inhibitor cocktail (Roche). On whole lysate, we evaluated caspase 9 protein levels (total and cleaved products), the ratio of phosphorylated and total MLC2, and ratio of phosphorylated and total ERK1/2. For analysis of mitochondrial and cytosolic proteins involved in apoptosis activation signaling pathway, subcellular protein fractions have been extracted using the ProteoExtract Subcellular Proteome Extraction kit (Calbiochem) following the manufacturer’s protocol.

For immunoblotting, 10 μg of protein was separated by 12 or 15 % SDS-polyacrylamide gel electrophoresis (PAGE) and subsequently transferred to a polyvinylidene difluoride (PVDF) membrane. Adequate transfer of protein was confirmed by Ponceau Red staining of the membranes. Antibodies against cytochrome c (Cytochrome C Releasing Apoptosis Assay Kit (#ab65311, Abcam), Bcl2 (#ab692, Abcam), Acetylcoenzyme A acyltransferase 2 (ACAA2) (#sc-100847, Santa Cruz Biotechnology), TIM23 (#611222 BD Biosciences), MitoProfile® total OXPHOS Rodent WB Antibody Cocktail (#ab110413, Abcam), myosin light chain 2 (MLC2) (#ab92721, Abcam), myosin light chain (pMLC2) (#ab2480, Abcam), Phospho-p44/42 MAPK (Erk1/2) (#4370, Cell Signaling), and p44/42 MAPK (Erk1/2) (137F5) (#4695, Cell Signaling) had been used.

In particular, MitoProfile® total OXPHOS Rodent WB Antibody Cocktail contains five mAbs, one each against CI subunit NDUFB8 (ab110242), CII-30 kDa (ab14714), CIII-Core protein 2 (ab14745) CIV subunit I (ab14705), and CV alpha subunit (ab14748) as an optimized premixed cocktail. The kit is suitable for western blotting analysis of the relative levels of the five OXPHOS complexes in mitochondrial preparations from mouse.

Equal protein loading and fractions purity were confirmed by probing for α-tubulin for whole lysate or cytosolic fraction and mitochondrial OXPHOS complex II protein for mitochondrial fraction.

Immunoreactive proteins were detected using ECL Plus (GE Healthcare) and quantified by the ImageJ 1.41 version software. At least, all samples were evaluated as duplicates. Representative western blots of all measured proteins are shown in Additional file 1: Figures S2-S4.

### Statistics

Data are shown as mean ± standard error of the mean (SEM). We only included animals with cannulation times less than 2 min and coronary flow between 1 and 4 ml/min at the end of the stabilization phase. Parameters were tested by one-way ANOVA followed by post hoc Dunnett comparing all treated groups with the untreated. For RPP, additionally we performed two-sided *T* test for independent samples. *p* values ≤0.05 were considered statistically significant.

## Results

### Pre-treatment with ERβ agonist and E2 improved LV function after I/R

**Fig. 1:**
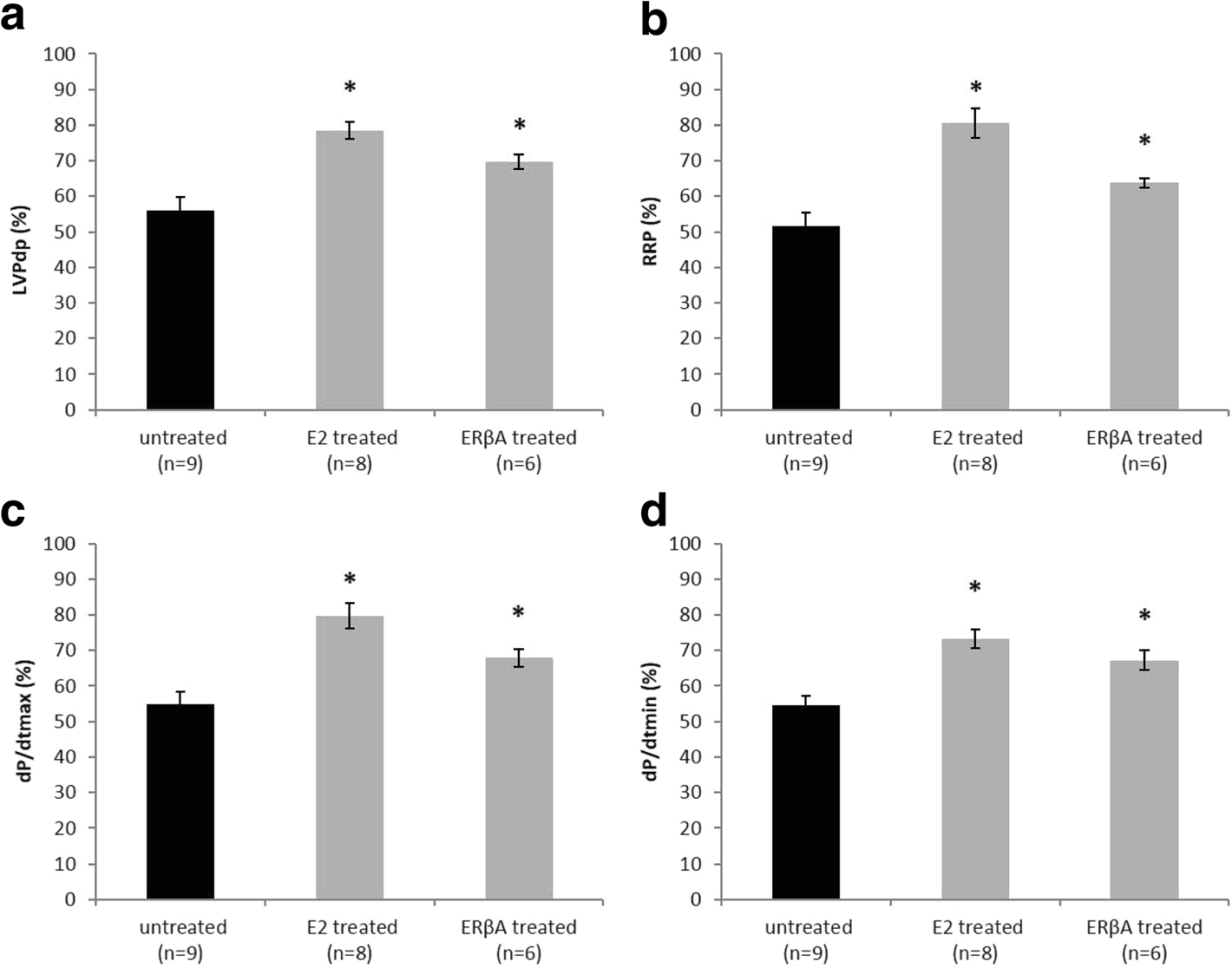
Left ventricular recovery after I/R LV developed pressure (LVPdp) (**a**). Rate pressure product (RPP) (**b**). dP/dtmax as marker of LV contractility (**c**) and dP/dtmin as marker for LV relaxation (**d**). Data are shown as mean ± SEM of parameters at the end of I/R in % of the start of ischemia. Significances were calculated by ANOVA followed by post hoc Dunnet and defined as significant with *p < 0.05

Developed left ventricular pressure (LVPdp) and rate pressure product (RPP) showed a significant better recovery in ERβA- and E2-treated groups compared with controls (Fig. 1a, b). Markers for contractility and relaxation, i.e., dP/dtmax and dP/dtmin, were significantly improved in the groups treated with ERβA or E2 (Fig. 1c, d). Heart rate (HR) was not changed significantly after I/R in all groups. All raw data are shown in the Additional file 1: Figure S1.

### Effect of ERβ agonist and E2 on necrosis and apoptosis after I/R

**Fig. 2:**
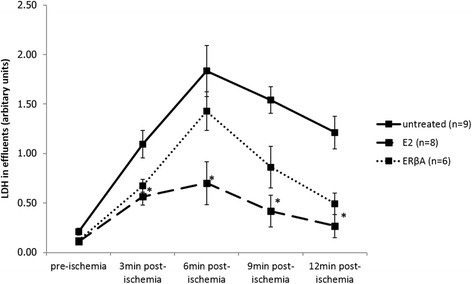
Measurement of LDH levels in effluents as marker for necrosis. Data shown are mean ± SEM at each time point. Significances were calculated by ANOVA followed by post hoc Dunnet for each time point and defined as significant with *p < 0.05

LDH in the effluent of isolated hearts, a marker of necrosis, was measured immediately before ischemia and 3, 6, 9, and 12 min immediately after start of reperfusion. All groups showed a peak in LDH levels between 3 and 6 min post-ischemia, which were significantly decreased only in the E2 group (Fig. 2). Pre-treatment with ERβA showed a similar effect that did not reach statistical significance.

**Fig. 3:**
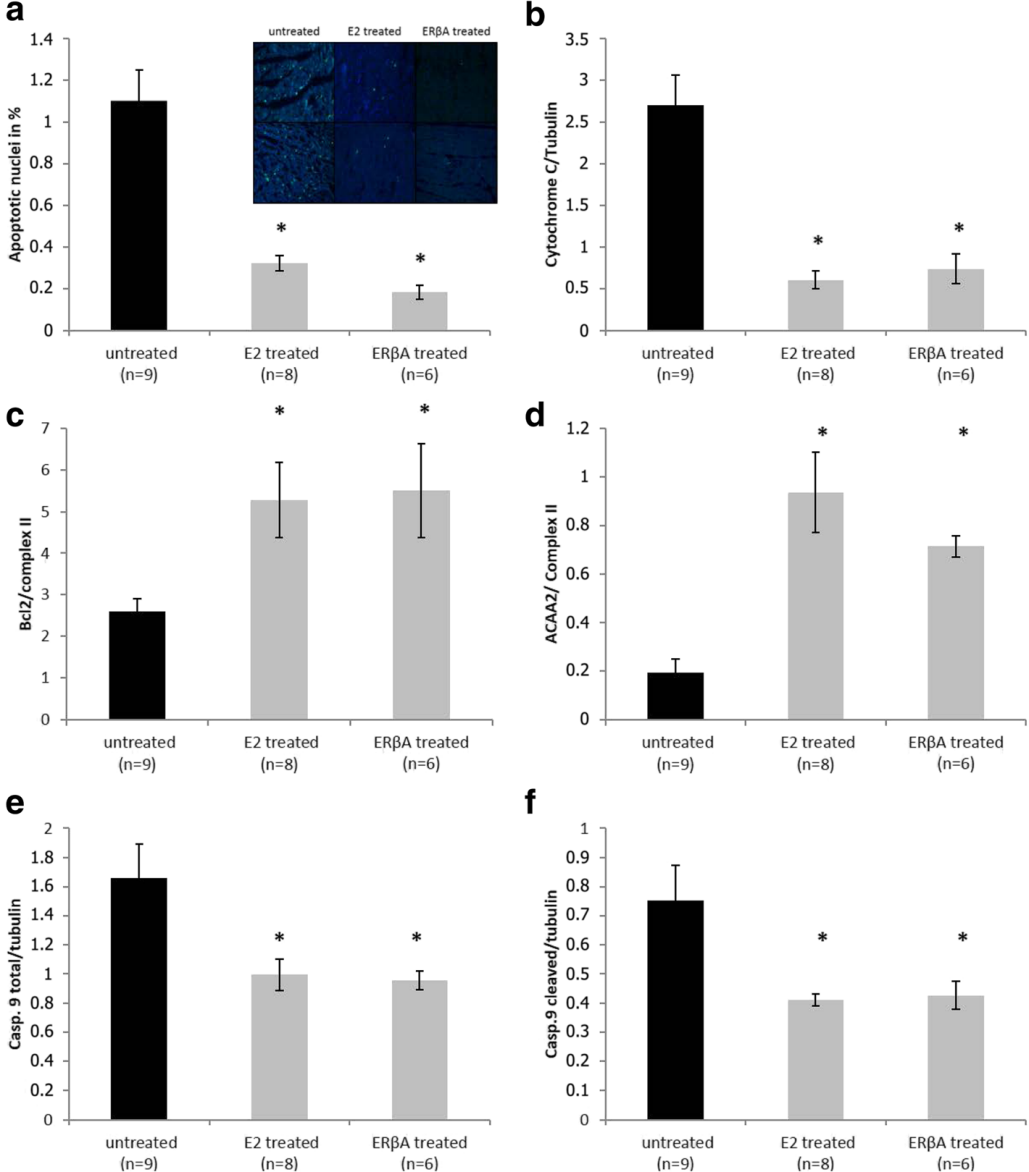
Apoptosis evaluation. Calculation of TUNEL-positive cells within the myocardium after treatment with E2 or ERβA (**a**). Representative pictures of TUNEL staining and relative protein expression to measure cytochrome c level in cytosolic fraction after treatment with E2 or ERβA (**b**). Relative protein expression data for measurement of Bcl2 (**c**) and ACAA2 (**d**) in mitochondrial fractions as well as total caspase 9 (**e**) and cleavage product (**f**) in whole cell lysate, Data are shown mean ± SEM. Significances were calculated by ANOVA followed by post hoc Dunnet and defined as significant with *p < 0.05

Assessing apoptosis, we found that the number of TUNEL-positive cells was significantly decreased by pre-treatment with ERβA or E2 (Fig. 3a). Cytochrome c release to the cytosol indicates mitochondrial damage and activation of the intrinsic pathway of apoptosis. ERβA, as well as E2 pre-treatment, significantly decreased cytochrome c levels in cytosolic fractions after I/R (Fig. 3b). Furthermore, the anti-apoptotic protein Bcl2 was increased in mitochondrial fractions of hearts pre-treated with ERβA and E2 (Fig. 3c). Expression levels of the predominant mitochondrial protein acetylcoenzyme A acyltransferase 2 (ACAA2) also known for its anti-apoptotic function [23] were higher in mitochondrial fractions from hearts treated with ERβA or E2 after I/R (Fig. 3d). Both ERβA and E2 pre-treatment decreased significantly the levels of caspase 9 and of its cleavage products in whole heart lysates (Fig. 3e, f).

### Mitochondrial integrity is improved by pre-treatment with ERβA or E2

**Fig. 4:**
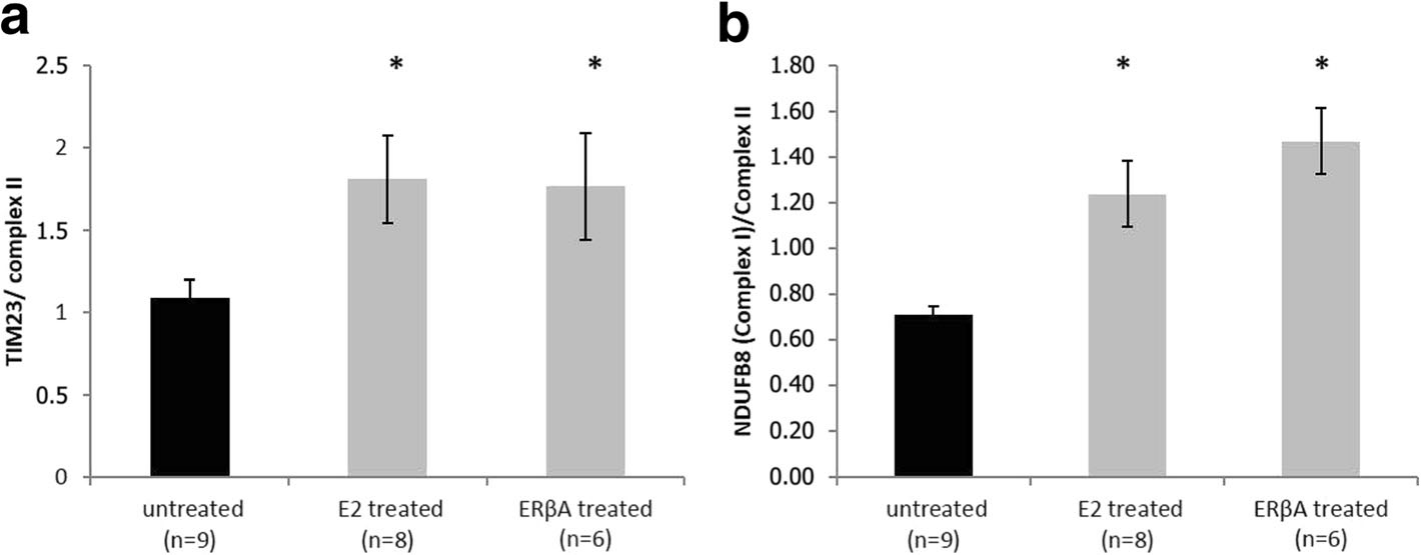
Evaluation of mitochondrial integrity. Relative protein level TIM23 (**a**) and NDUFB8 of OXPHOS complex I (**b**) in mitochondrial fractions of LV protein lysates. Data shown are mean ± SEM. Significances were calculated by ANOVA followed by post hoc Dunnet and defined as significant with *p < 0.05

To assess integrity of the mitochondrial membrane structure, we also evaluated the effects of ERβA and E2 pre-treatment on the mitochondrial translocase TIM23 that is located in the inner mitochondrial membrane. We observed significant higher levels of TIM23 protein in mitochondrial fractions of ERβA- and E2-treated groups than in controls (Fig. 4a). Furthermore, the levels of complex I protein of OXPHOS chain in mitochondrial protein fractions in both treated groups (*p* < 0.05) were significantly higher than those in untreated controls (Fig. 4b). Together with the reduced cytochrome c release, these data suggest a better maintenance of mitochondrial integrity and respiratory function in the ERβA- and E2-treated groups.

### Myosin light chain 2 phosphorylation by E2 and ERβA pre-treatment

**Fig. 5:**
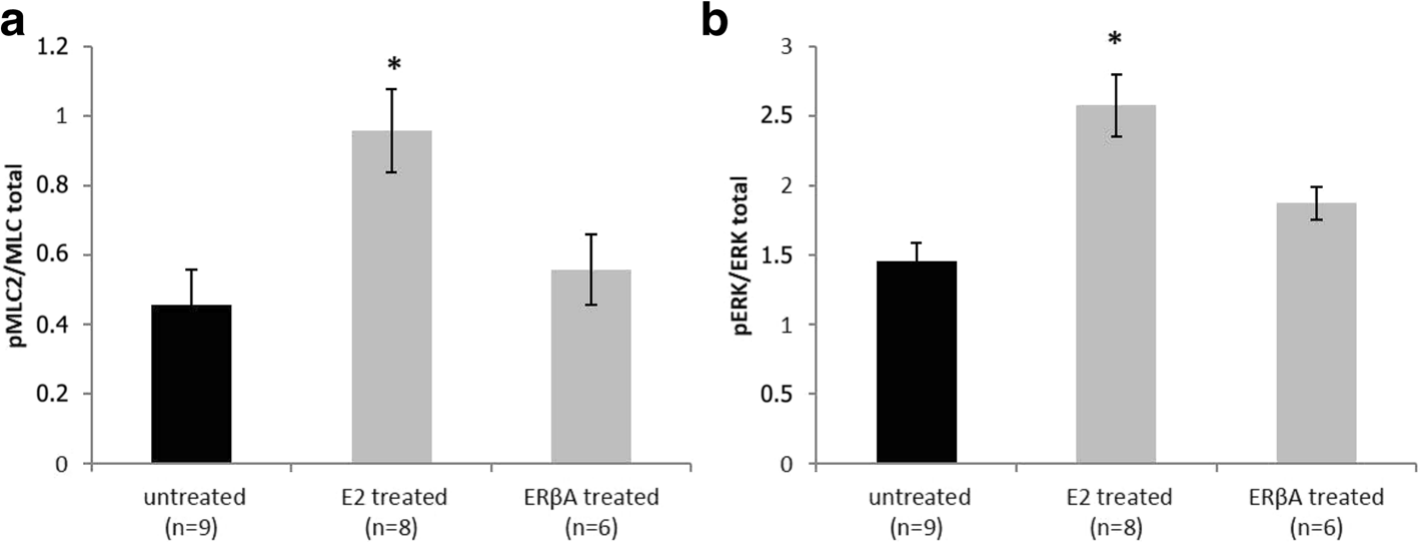
Modulation of contractility. Relative protein phosphorylation of MLC2 (**a**) and of ERK1/2 (**b**). Data shown are mean ± SEM. Significances were calculated by ANOVA followed by post hoc Dunnet and defined as significant with *p < 0.05

Phosphorylation status of MLC2 alters Ca^2+^ sensitivity of myofilaments and is therefore important for cardiomyocyte contractility [24, 25]. We measured total MLC2 and phosphorylated levels of MLC2 (pMLC2) as marker for contractile function in total protein lysates of LV after I/R. We observed a significant greater degree of MLC2 phosphorylation in the E2-treated group in comparison with controls, which was not found in ERβA-treated animals (Fig. 5a). ERK1/2 is located upstream of MLC2 and can phosphorylate MLC2. E2- but not ERβA-treated mice exhibited a significant higher level of phosphorylation of ERK1/2 than controls (Fig. 5b). These data suggest that E2 but not ERβA improves myocyte contractility.

## Discussion

The present study shows that activation of ERβ, as well as E2 treatment, prior to I/R attenuates mitochondrial damage and cell death and improves cardiac recovery. Here we show for the first time that a specific ERβ agonist reduces mitochondria-dependent apoptosis and contributes to the maintenance of mitochondrial integrity after ischemia and reperfusion injury. Furthermore, we found an ERβ-independent effect of E2 on MLC2 phosphorylation that may contribute to better cardiac contractile recovery under I/R conditions.

Although E2 in various experimental models induces the improvement of functional cardiac recovery after I/R, the pathways involved and the relative contribution of ER subtypes are still a matter of debate. Reduction of infarcted necrotic area and improvement of functional indexes recorded in Langendorff experiments already suggested that E2 can maintain myocardial tissue viability through reduction of necrosis and apoptosis [7, 15, 26] and was reproduced in our system. Reduced LDH release in the first 12 min of reperfusion, indicating early necrosis, was reduced by E2 treatment, but not by ERβA, indicating that ERα or GPER may also have conferred cardioprotection. Of note, in our system, a specific ERβ agonist reduced apoptosis and led to the preservation of mitochondrial integrity which was associated with a better cardiac recovery after I/R compared with the untreated.

Apoptosis activation after I/R is driven by the loss of mitochondrial stability leading to mitochondrial release and cytosolic activation of cytochrome c [17]. These events result in mitochondrial dysfunction and cytosolic caspase activation, which contribute to myocardial contractile dysfunction, necrosis, and apoptosis after reperfusion. In particular, we found that both ERβA and E2 increased the amount of the anti-apoptotic protein Bcl2 in the mitochondria.

Contrastly, in a study with prostate cancer tissue from men, the treatment with the ERβ agonist DPN resulted in a significant induction of apoptosis [27]. These contrary effects of specific ERβ activation can be explained by the sex-, cell-, and tissue type-specific regulation of estrogen receptors and its pathways. For example, our group could show in a study with male and female wild-type and ERβ-deficient mice that sex differences in the development of heart failure are related to sex-specific actions of ERβ. In that study, ERβ promoted fibrosis in male hearts but inhibited fibrosis in females [28]. Physiological relevance of our recent finding is supported by previous studies showing that increased Bcl2 levels are associated with reduced apoptosis, reduced infarct size, and improved recovery of cardiac function after I/R [29, 30]. Moreover, expression of Bcl2 is controlled by ERβ. Bcl2 family proteins, located in mitochondrial membranes, provide protection against pro-apoptotic stimuli [17]. Bcl2 prevents permeabilization of the outer mitochondrial membrane [31] and thereby prevents the release of cytochrome c from mitochondria. Accordingly, we found lower cytochrome c levels in the cytosol of ERβA- and E2-treated hearts than in controls after I/R. The downstream apoptotic pathway induced by cytochrome c release from the mitochondria is the cytosolic recruitment and activation of caspase cascade in the apoptosome [17]. Accordingly, we found a significant downregulation in total and cleaved caspase 9 in E2- and ERβA-treated groups suggesting that E2 via ERβ can modulate caspase 9 through two mechanisms: a direct inhibition of pro-caspase 9 synthesis and an indirect effect through a decrease in caspase 9 activation by cytochrome c. Since caspase inhibitors reduce infarct size [32, 33], ERβ modulation of caspases could be cardioprotective.

In ERβA- and E2-treated groups, we also found higher protein levels of ACAA2. ACAA2 is a mitochondrial enzyme involved in lipid metabolism that was identified as a binding partner for the pro-apoptotic protein Bcl2 and 19-kDa interacting protein-3 (BNIP3) [23, 34]. ACCA2 can abolish BNIP3-mediated apoptosis and mitochondrial damage in hepatic and osteoclastic cell lines [23]. It is likely that similar mechanisms might occur in cardiomyocytes in response to I/R. We therefore conclude that ERβ reduces mitochondria-related apoptosis through several mechanisms.

Mitochondria in the ERβA and E2 pre-treated groups had higher levels of TIM23, a marker of integrity of the mitochondrial inner membrane [35]. This is the first time that an interaction between TIM23 and E2/ERβ signaling in cardiac damage due to I/R is reported. This increase in TIM23 protein levels together with the reduced cytochrome c release and the higher expression of anti-apoptotic proteins suggests that E2 induced stabilization of mitochondrial structure and integrity by activating ERβ.

The respiratory chain in the inner mitochondrial membrane is responsible for generating ATP and consists of five major membrane protein complexes. We found here that the pre-treatment with ERβA and E2 increases the protein levels of mitochondrial complex I, supporting the notion that E2 reduces I/R damage through an ERβ-dependent mechanism.

Other mechanisms could contribute to the better contractile recovery after I/R in ERβA and E2 pre-treated hearts. Our group previously reported that E2 treatment can modulate myocardial contractility via MLC2 function in a sex-specific manner [21]. We observed an increase in phosphorylation of MLC2 in E2- but not in ERβA-treated groups. Since ERK1/2 acts upstream of MLC2 leading to its phosphorylation [36], we assessed ERK1/2 phosphorylation and found that it was increased only in the E2-treated group. These findings suggest that E2 leads to MLC2 phosphorylation by an ERβ-independent mechanism.

This study has several limitations. Most importantly, we did not confirm the specificity of the ERβA. This has however been well established in previous investigations [8]. We limited our study to a single dose of ERβA and E2, which does not exclude varying effects at different doses. We also used females only. Since we have previously shown sex-specific effects of E2 in cardiac cells and tissues [21, 22, 28, 37], it will be interesting to assess the effects of ERβ activation under I/R also in males.

## Conclusions

The present study suggests that the activation of ERβ by E2 or a specific agonist essentially contributes to a reduction of cardiac stress by reducing apoptosis and preservation of mitochondrial integrity, and improved functional recovery after I/R. The identification of new ERβ-mediated E2 effects opens new opportunities to therapeutically targeting these pathways in different clinical settings.

## Additional file

Additional file 1: Figure S1. Raw data of cardiac function during ischemia and reperfusion. Data are shown as mean ± SEM. Significances were calculated by ANOVA followed by post hoc Dunnet and defined as significant with *p* < 0.05. Figure S2: Representative western blots of ACAA2, Bcl2, TIM23, NDUFB8 and cytochrome c in cytosolic (F1) and mitochondrial (F2) fractions and corresponding controls (tubulin for F1, complex II for F2). Figure S3: Representative western blots of whole tissue lysates for caspase 9, caspase 9 cleaved and corresponding loading controls (tubulin and GAPDH). Figure S4: Representative western blots of whole tissue lysates for MLC/pMLC and ERK/pERK. (DOCX 876 kb)

## Abbreviations

I/R: Ischemia/reperfusion
E2: 17β-Estradiol
ERβA: Estrogen receptor β agonist
LDH: Lactate dehydrogenase
