# Supplementary material for: Reduction of apoptosis and preservation of mitochondrial integrity under ischemia/reperfusion injury is mediated by estrogen receptor β
Source: Biol Sex Differ. 2016 Sep 23;7:53. doi: 10.1186/s13293-016-0104-8 (PMC5035458; doi:10.1186/s13293-016-0104-8)
Supplement: Additional file 1: Figure S1. — Raw data of cardiac function during ischemia and reperfusion. Data are shown as mean ± SEM. Significances were calculated by ANOVA followed by post hoc Dunnet and defined as significant with p < 0.05. Figure S2: Representative western blots of ACAA2, Bcl2, TIM23, NDUFB8 and cytochrome c in cytosolic (F1) and mitochondrial (F2) fractions and corresponding controls (tubulin for F1, complex II for F2). Figure S3: Representative western blots of whole tissue lysates for caspase 9, caspase 9 cleaved and corresponding loading controls (tubulin and GAPDH). Figure S4: Representative western blots of whole tissue lysates for MLC/pMLC and ERK/pERK. (DOCX 876 kb) [file 13293_2016_104_MOESM1_ESM.docx]

**Additional file 1**

**
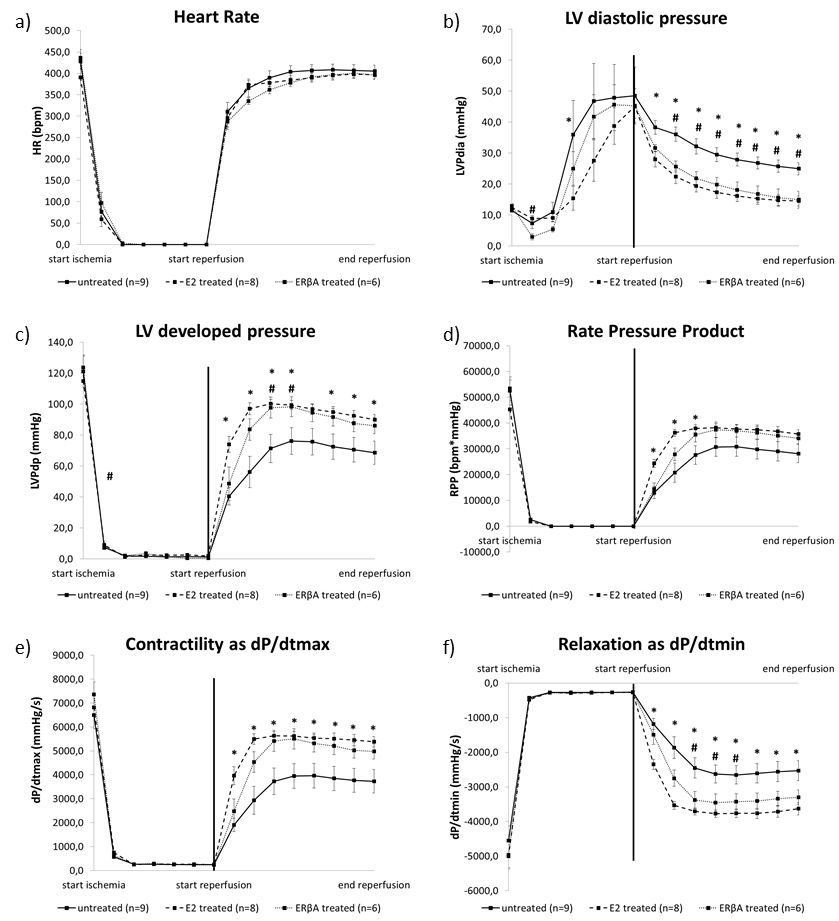
**

**Figure S1:** Raw data of cardiac function during ischemia and reperfusion. Data are shown as mean ± SEM. Significances were calculated by ANOVA followed by post-hoc Dunnet and defined as significant with p<0,05.


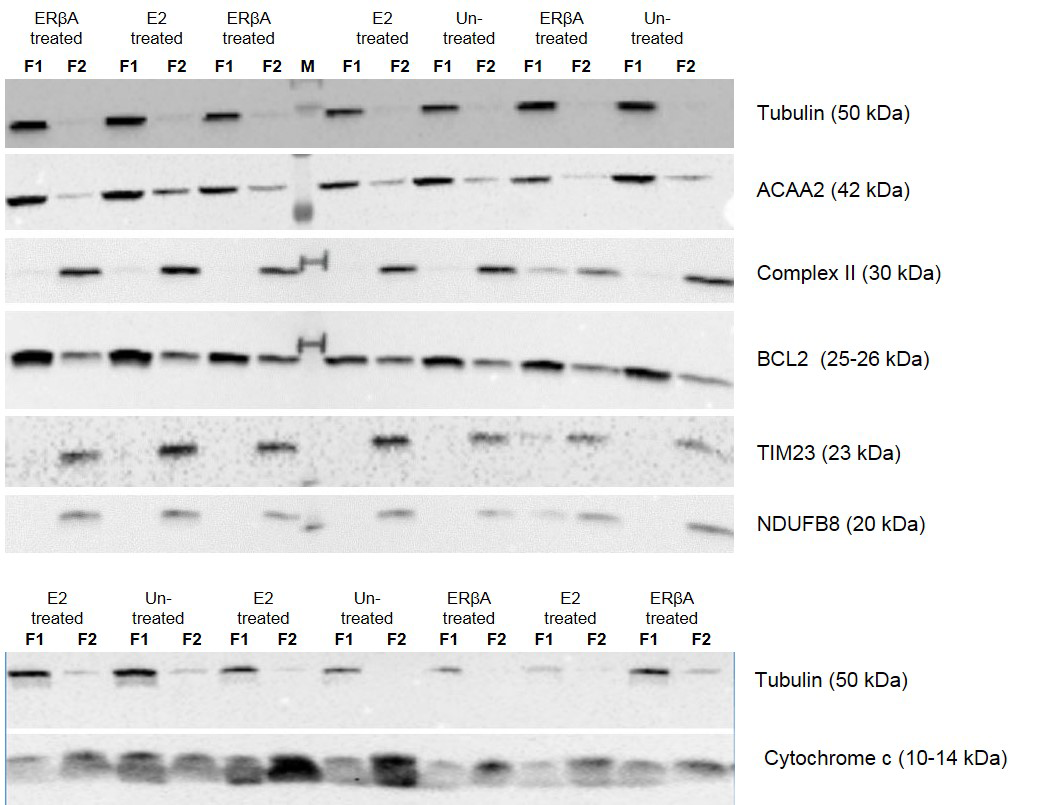


**Figure S2:** Representative western blots of ACAA2, Bcl2, TIM23, NDUFB8 and cytochrome c in cytosolic (F1) and mitochondrial (F2) fractions and corresponding controls (tubulin for F1, complex II for F2).


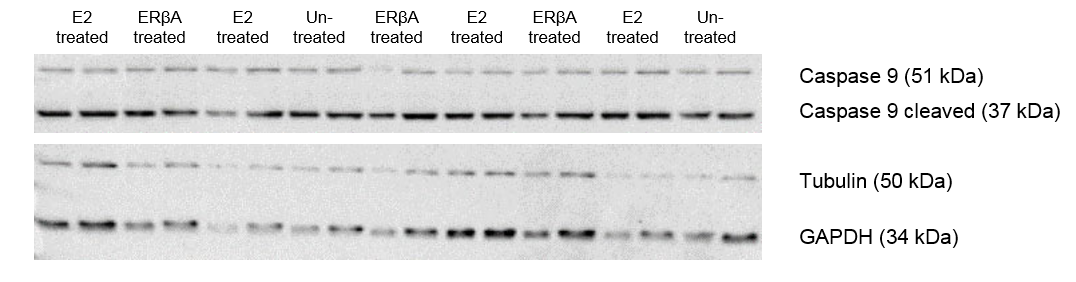


**Figure S3:** Representative western blots of whole tissue lysates for caspase 9, caspase 9 cleaved and corresponding loading controls (tubulin and GAPDH).


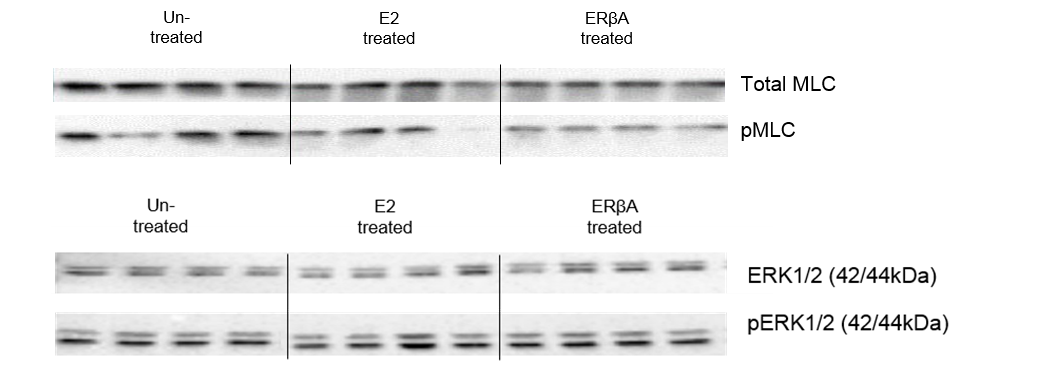


**Figure S4:** Representative western blots of whole tissue lysates for MLC/pMLC and ERK/pERK
